# Human rights, public health and medicinal cannabis use

**DOI:** 10.1080/09581596.2015.1038218

**Published:** 2015-11-16

**Authors:** Melissa Bone, Toby Seddon

**Affiliations:** ^a^ School of Law, The University of Manchester, Manchester, UK

**Keywords:** drug policy, human rights, medical cannabis

## Abstract

This paper explores the interplay between the human rights and drug control frameworks and critiques case law on medicinal cannabis use to demonstrate that a bona fide human rights perspective allows for a broader conception of ‘health’. This broad conception, encompassing both medicalised and social constructionist definitions, can inform public health policies relating to medicinal cannabis use. The paper also demonstrates how a human rights lens can alleviate a core tension between the State and the individual within the drug policy field. The leading medicinal cannabis case in the UK highlights the judiciary’s failure to engage with an individual’s human right to health as they adopt an arbitrary, externalist view, focussing on the legality of cannabis to the exclusion of other concerns. Drawing on some international comparisons, the paper considers how a human rights perspective can lead to an approach to medicinal cannabis use which facilitates a holistic understanding of public health.

## Introduction

Attempts to reconcile human rights and public health frameworks highlight ‘embedded tensions between the individual and the collective’, since human rights regimes are individualistic, as opposed to centring upon whole populations (Labonté, 2008, p. 478). Whilst the concepts of public health and human rights are complex, this paper advances the notion that both regimes are, in fact, complementary. It will demonstrate how a human rights lens can better inform public health policies relating to medicinal cannabis use and psychoactive substance use more generally. Both human rights and public health relate to the regulation of State behaviour through international norms. The lens of human rights can offer a distinctive and useful perspective when contemplating medicinal cannabis use, since drug policy is inherently political. There exists a key tension between a State’s interests in restricting certain psychoactives (the justification usually being to protect the health, welfare, morality and/or safety of its citizenry) and an individual’s rights to consume them, in order to regain control over their own faculties and bodily integrity. Drawing upon biological and cultural evidence of psychoactive use throughout human history, Siegel (2005, p. 15) eloquently describes the conflict as follows: ‘The human pursuit of intoxication is motivated by a strong biological drive that pits individual needs against those of society’.

Whilst there is no obvious way to resolve this core tension between individual and collective interests, this paper will argue that the lens of human rights can offer a distinctive and useful perspective, as these rights are designed precisely to address this conflict through their predominant focus on the vertical relationship between States and their subjects (Donnelly, 2003; Nickel, 2010). Moreover, most human rights are derogable as they strive to protect and respect individual autonomy, but not in ways that allow for the rights of others to be infringed (Perry, 1998). Political engagement is likewise at the crux of public health issues (Coggon, 2012). For Coggon (2012), allocating State responsibilities is contestable, since States have legal duties to provide environments in which humans can flourish (see also Powers & Faden, 2006), whilst simultaneously respecting and protecting citizens’ rights to be left alone. Nevertheless, as Tsakyrakis (2009) argues, if employed correctly, human rights should be able to provide a balance through articulating and openly debating the moral considerations involved. To examine how human rights can address and alleviate the deep-rooted political tension between the State and the individual in the drug policy sphere, this paper will consider the human right to health. It will predominately focus upon a leading UK medicinal cannabis case – drawing also on some international comparisons – to explore critically how a human rights lens can inform the development of public health policies relating to medicinal cannabis use.

## Concepts of ‘health’ and ‘public health’

Whilst a human right to health is not overtly recognised legally in the UK, it is protected internationally (Article 12, International Covenant on Economic, Social and Cultural Rights). Moreover, a human right to health can be incorporated domestically under the auspices of core civil and political rights contained within the European Convention on Human Rights (1950) (ECHR). Human rights to life and privacy (Articles 2 and 8, ECHR) enable more generalised human rights to health and to non-interference from the State. The United Nations drug conventions are also designed to provide certain psychoactive substances for medical and research purposes, thereby facilitating commodity control as well as penal control (Boister, 2001). Whilst *rights* to health may be evident, defining the term ‘health’ itself can be rather more elusive. From the Hippocratic Oath (Edelstein, 1943), to the World Health Organisation (WHO) definition (1948/1998), the notion of what is ‘health’ and what constitutes ‘good health’ is an idea which has been grappled with for centuries. In broad terms, definitions of health can be categorised as either medicalised or social constructionist. Medicalised definitions hold that diseases are largely invariant over time and place (Boorse, 1975). Those who favour a medical model of health can be described as *realists*, as they consider that health and disease are ubiquitous features of the natural world and draw upon descriptive definitions of health which are based upon biological function (Boorse, 1975). Social *constructionist* definitions, on the other hand, distinguish disease (the biological condition) from illness (the social condition), on the basis that the meaning and the experience of illness are shaped by social and cultural systems and the environment in which we live (see Conrad & Barker, 2010). The social constructionist definition does not solely draw upon our biology or have a materialistic basis; rather, it accepts that ‘health’ cannot be understood in isolation from the wider social and cultural context.

According to Coggon (2012), these varied contextualisations of what ‘health’ is and how we should understand it can lead to a crucial tension in determining what is ‘good’ or ‘healthy’ for us. He states that there exist externalist and internalist views on what ‘health’ means, representing, respectively, state-imposed vs. individually determined notions. In general, the medical establishment and policy-makers commit to a more medicalised and externalist view on what they believe to be ‘good’ for us within the drug policy field, which can leave the individual to grapple with an internalist view on what they believe to be ‘good’ for themselves. Definitions of public health similarly allow for broad contextualisations and reiterate the tensions Coggon identifies. The overarching concern of public health with populations and the total system of health necessitates a consideration of the political duties which conjoin communities, and an evaluation of how much power the State should wield in order to maximise health benefits and mitigate harms within populations (Coggon, 2012). For Sen (2004), the notion of public health can alleviate crucial tensions in substantiating what is ‘good’ for us, as he defends both internal and external views on health, advocating a combined approach. He observes that the two viewpoints should coexist in order to avoid an overly limited or detached outlook, as the potential for discrepancies between them usefully highlights and makes visible the political question of precisely *who* should be the arbiter in health policy debates (see Coggon, 2012, p. 215). In this sense, the well-known WHO (1948/1998) definition of health is helpfully broad: ‘a state of complete physical, mental, spiritual and social well-being and not merely the absence of disease or infirmity.’

The WHO definition’s aspirational and utopian aims are well suited to human rights. Through combining social constructionist and medicalised definitions, by focusing on well-being and health promotion as well as reducing disease, it can also challenge the status quo, and the legalistic focus, within drug policies**.** Whilst there is currently a significant debate in the UK and internationally between those wishing to maintain the policy of prohibition and those arguing for drug policy reform (Bone, Mungroo, & Ping, 2014), in relation to illicit substances the primary focus is on the legality of a substance, often to the exclusion of everything else. As we will see, the leading medicinal cannabis case in the UK exemplifies how the State can adopt an externalist view, focusing on what they believe to be good for individual citizens, disregarding both the medical evidence which considers the therapeutic application a controlled substance may have, and any internalist accounts of how an illicit substance could be beneficial for individual health**.** Comparisons will be made internationally, and by employing a human rights perspective, the case law will act as a useful starting point to advance public health policies for medicinal cannabis which embrace broader conceptualisations of health.

## Medicinal cannabis and the human right to health: the *Quayle* case and international case law

*R v. Quayle; R v. Wales; R v. Taylor and another; R v. Kenny; Attorney General’s Reference (No. 2 of (* *2005* *)* (hereafter, *Quayle*) is the leading UK case on the right to use cannabis medicinally. It involves a group of cases heard conjointly by the Court of Appeal. All of the individuals suffered from a range of medical conditions, including (but not limited to) phantom limb pain, multiple sclerosis, AIDS and pancreatic cancer, and they tried to alleviate associated pain and other symptoms through using cannabis. The majority were convicted at trial under the Misuse of Drugs Act, 1971 (hereafter the MDA), for either possession, possession with intent to supply, or cultivation. The individuals wished to challenge their original verdicts through arguing that the prohibitions on their right to self-medicate infringed their human rights. Concurrently, the Attorney General was appealing a case whereby one defendant, Mr Ditchfield, was acquitted at his trial, as the jury accepted his defence of medical necessity for supplying cannabis to multiple sclerosis and cancer sufferers. Therefore, as well as the human rights arguments, Quayle and others were additionally appealing against the decision of their trial judge not to put the medical necessity defence before a jury. The judges dismissed all of the appeals and agreed with the Attorney General that the defence of medical necessity was not applicable to those who wished to supply cannabis to others.

By focusing on whether the cannabis use was necessary and was the only means by which the defendants could alleviate their conditions, the judicial reasoning failed to challenge the status quo and whether it was in fact the prohibition of cannabis which was wrong through infringing individual autonomy and human rights to health. Nevertheless, the appellants still sought to rely upon Article 8(1) in the ECHR to argue that an absolute prohibition of cannabis infringed their human rights to self-medicate. Article 8(1) provides that: ‘Everyone has the right to respect for his private and family life’. The Court of Appeal held that whilst Article 8(1) could be infringed in relation to those self-medicating with cannabis, any infringements would be justified due to the limitations contained within Article 8(2) ECHR. Such limitations allow the court to restrict an individual’s human right where it is deemed to be necessary in order to protect (amongst other interests): public safety, public health, morals, or the rights and freedoms of others. However, there was a complete refusal to analyse the grounds on which the court was able to limit the defendants’ Article 8 rights. The issue was simply deferred to parliament in line with Beck’s (2008) observation that the judiciary rarely wish to challenge the executive in politically sensitive areas. We might, however, feel entitled to expect that when a State makes a claim about protecting its citizenry, it should be required to thoroughly analyse exactly what it is that is being protected and to specify the justification for restricting a human right. Whilst superficially the limitations are employed on a ‘harm-to-others’ rationale, the fact that the supposed harms fail to undergo any empirical analysis exemplifies the circumscribed nature of the judicial balancing process. What is more, the judiciary made reference to the Runciman report (the Police Police Foundation, 2000) and that committee’s conclusions that the therapeutic benefits an individual could derive from cannabis outweighed the risks to themselves or society, yet they failed to analyse the conclusions drawn. Instead, they consistently made reference to the fact that they were required to operate within the prevailing ‘legislative scheme’ (*Quayle*, p. 3643; paras. 54, 67). The judges therefore adopted a narrow reasoning process when considering the health rights of medicinal cannabis consumers, an approach which has been followed consistently by the UK judiciary (e.g. see *R v. Altham,* 2006).

The international cases on medicinal cannabis use present a rather different story. The separation of state and federal political and legal systems, particularly within the US, has facilitated diverse regulatory and judicial approaches. At the time of writing, 23 US states and Washington DC have legalised and regulated medicinal cannabis to varying degrees. A growing number of other jurisdictions, including Canada, the Netherlands, Israel and the Czech Republic, also have provisions legalising and regulating cannabis for medicinal purposes (Rolles & Murkin, 2013). In the US, activists first exploited the legal ‘space’ between state and federal laws through the enactment of Proposition 215 or the Compassionate Use Act (1996). This Act allowed doctors in California to recommend cannabis to patients, where primary caregivers would possess and cultivate it for the consumer’s personal medical use. Other states followed suit, thus facilitating a de facto legal foundation for various regulatory models such as cannabis social clubs or dispensaries. This has left the legal position in some tension between the state and federal levels. In 2003, the US Supreme Court agreed with an appeal court ruling that threats from the federal government to revoke doctors’ licences based on their medical cannabis recommendations violated core privacy rights, protected by virtue of the privileged status of the doctor/patient relationship (*Conant v. Walters,* 2003). This decision resolved the tension between State and individual interests by approving this special relationship, as a doctor’s duty to the patient should not be sacrificed to avoid prosecution from the federal government. The California Court of Appeal decision in *City of Garden Grove v. Superior Court (* 2007) similarly accords strong weight to state regulations, since the police department was ordered to give the defendant his cannabis back stating that ‘because the act is strictly a federal offense, the state has no power to punish [...] it [...] as such’.

Despite this, the US Supreme Court has also upheld the ability of federal officials to enforce federal law which conflicts with state law. In *US v. Oakland Cannabis Buyers’ Cooperative* (2001) and *Gonzales v. Raich* (2005), the courts failed to accord any weight to individuals’ autonomy and ignored the social benefits of how cannabis cooperative systems alleviate access hardships faced by patients, by simply deferring to the legislature, in a similar manner to *Quayle.* In contrast, in the Canadian case of *R v. Parker* (2000), which concerned the defendant’s use of cannabis to control his epilepsy, the incompatibility between the right to health protected under s.7 of the *Canadian Charter of Rights and Freedoms* and the prohibition of cannabis cultivation and possession under the Controlled Drugs and Substances Act 1996 was successfully challenged as contrary to ‘fundamental justice’. Moreover, Canadian applicants additionally challenged the constitutionality of their Marihuana Medical Access Regulations (MMAR) in 2003, on the grounds that medicinal consumers had no legitimate access to cannabis, despite legal exemptions allowing for its use (*Hitzig v. Canada,* 2003). The Ontario Superior Court declared that the regulations were unconstitutional; consequently, Health Canada is now able to dispense cannabis to medicinal consumers. Thus, the judicial process both reflects and constitutes the laws and policies relating to medicinal cannabis. Though the international case law is inconsistent, a human rights perspective arguably sheds light on how formalised and informalised regulatory models can develop. These international comparisons also suggest how the UK’s approach to health in this context has been unduly narrow and rigid.

## Critiquing the case law: developing public health policies for medicinal cannabis from a human rights perspective

Through focussing on the legality of cannabis, the UK’s Court of Appeal in the *Quayle* case failed to analyse any medical evidence and adopted what the political philosopher Buchanan (2008) might term a parochialist view, in the sense that it implicitly rested on an unqualified acceptance of the moral–political status quo of cannabis prohibition. The judges in *Quayle* argued that parliament and the elected government of the day were better placed to determine ‘social, medical and legislative policy’ (para. 68), because for the court to do so ‘would involve an evaluation of the medical and scientific evidence’ (para. 68), and ‘a greater understanding of the nature and progress of the tests of cannabis which have taken and are taking place’ (para. 68). But, as Buchanan (2008) observes, the correct application of human rights norms should involve drawing upon reliable, factual and relevant information to guard against parochialism and an arbitrarily restricted set of moral values. Unfortunately, the *Quayle*, *Oakland Cannabis Buyers’ Cooperative* and *Gonzales* cases reflect a clear desire to maintain the prohibitive status quo. The *Quayle* judgment in particular failed to conform to even the most basic human rights adjudication process, which according to Alder (2006) involves embracing consequentialist as opposed to deontological reasoning, as the protection of rights typically hinges on assessing outcomes. For Alder, this process lends itself more easily to a cost-benefit analysis, rather than a more abstract consideration of the intrinsic value attached to an individual’s rights. Though this observation is intended to criticise the inherent artificiality when balancing two incommensurable values in human rights disputes, that is, a State’s interests against an individual’s, even this failed to emerge in *Quayle*.

There was in fact room in the case for a detailed discussion of the medicinal uses of cannabis. In *Quayle,* there were nine instances where the Court of Appeal referred to the expert evidence adduced from earlier judgments, which concerned the medical benefits of cannabis consumption, and there was one discussion of the risks involved. Furthermore, pharmaceutical interest in the therapeutic application of various cannabinoids was increasing around this time. GW Pharmaceuticals have now successfully licensed their ‘cannabinoid medicine’, *Sativex*, in 11 countries (including the UK), for the treatment of multiple sclerosis spasticity (GW Pharmaceuticals, 2014). This interest, combined with the substantial historical research literature (Aldrich, 1997; Russo, 2005, 2007) and more recent evidence on the medicinal usage of cannabis (Guzman, 2003; Leung, 2011), especially in the treatment of cancer (see ‘Canadians for Safe Access’, n.d. http://safeaccess.ca/research/), means that in view of Buchanan’s reasoning, such uses at least warranted a discussion. What is more, several countries hold licences to prescribe *Bedrocan* (pharmaceutical grade medicinal cannabis) to treat a wider array of medical conditions (Bedrocan Canada, 2014; n.p.). The company first produced medicinal cannabis in the Netherlands through their Office of Medicinal Cannabis government-run programme. This Dutch model was unique as it dispensed a variety of cannabis strains to medicinal consumers through pharmacies, depending upon their health needs. This programme has a well-documented source of data, since consumers typically purchase their product through the same pharmacy as any other prescription medicine (Hazekamp & Heerdink, 2013). Indeed, the company has been hailed as promoting quality control, education, research and patient care, and it is perhaps for this reason that several other countries hold import licences (Hazekamp & Heerdink, 2013). Most recently, *Bedrocan* has been licensed in Canada (Bedrocan Canada, 2014), which may help to begin to address the lack of strain variety in Health Canada’s centralised medicinal cannabis programme. The public health implications of providing a choice of cannabis strains are arguably substantial, since Di Forti and Murray (2014) and Curran (2014) have acknowledged the potential negative impact that higher tetrahydrocannabinol cannabis strains could have upon the mental health of some consumers. As Nutt, King, and Nichols (2013) argue, the UK Misuse of Drugs Regulations (2001) currently make it very difficult to conduct appropriate scientific research on different cannabis strains, as cannabis is listed in schedule 1 of the Regulations, meaning that it is subject to the tightest controls. They suggest that this is adversely and unnecessarily constraining the ability of clinical researchers to explore the considerable therapeutic potential of cannabis (see also Nutt, 2015).

The ever-increasing medicinal developments have also impacted upon individuals who embrace a more internalist conception of health. According to Hayry (2004), Tsakyrakis (2009) and Walsh (2010), an individual’s values and beliefs should form an intrinsic part of any judicial reasoning process. To interfere with a person’s health choices without any sound ethical reasoning invites a determinist response (Walsh, 2010) which thereby fails to adequately balance the interests of the State with those of the individual. If the judiciary in *Quayle*, *Oakland Cannabis Buyers’ Cooperative* or *Gonzales* accepted any internalist deliberations, then a bona fide balancing act may have been conducted, which explored the therapeutic benefits of empowering individuals to make their own health care choices. Coomber, Oliver, and Morris (2003), Jones (2006) and Waldstein (2010) all observe that self-care practices in relation to medicinal cannabis use are increasing. The organisation ‘Bud Buddies’, founded by an original *Quayle* defendant, Jeff Ditchfield, have illegally supplied medical cannabis to others for 15 years, and they have been heavily influenced by Guzman’s scientific research into the efficacy of cannabis oil for treating cancer. One of the ‘Bud Buddies’ patients featured in their ‘Project Storm’ film acutely summarises his position: ‘It’s better to be a living cannabis criminal, rather than a dead law-abiding citizen’ (n.d. http://projectstormthefilm.com). This powerful testimonial supports Sen’s (2004) view that one should be wary of public health policies which only seek to protect plural, externalist conceptions of what is ‘good’ for us. In fact, one of the primary criticisms directed at Health Canada in the *Hitzig* case was their refusal to legalise cannabis compassion clubs or to create properly regulated distribution centres. Lucas (2008) observes that Canada’s compassion clubs and societies supply more medicinal cannabis, produce a more varied supply and participate in more medical research than the government’s programs, all at no cost to the taxpayer. In this sense, the claim in the *Quayle* judgment that whilst Article 8(1) could apply to those self-medicating with cannabis, it would definitely *not* extend to those involved in supply appears to be too narrow from a public health perspective.

This unyielding judicial restriction in *Quayle* prevented the court from fully engaging with the situation of two of the appellants in the case, Taylor and Lee, who ran a holistic clinic which supplied cannabis to patients, many of whom were suffering from debilitating diseases or terminal illnesses. As Szasz famously observed in his polemic, *Ceremonial Chemistry*, ‘today in therapeutic societies, only the physician is allowed to dispense ‘dangerous drugs’ [..I]f anyone else does so, he is called a ‘pusher’, and is again condemned and punished regardless of the consequences of his efforts’ (Szasz, 2003, p. 66). Yet, through embracing a broader conception of health, as facilitated by a human rights lens, the judiciary could have feasibly recognised the preferential treatment afforded to the pharmaceutical industry and the dominant health care systems. If the UK government were to officially recognise this state of affairs, public health policies could develop which reflect the de facto social club and compassionate club models observed in the US and Canada (Lucas, 2008; Reiman, 2008). When researching medicinal cannabis facilities in California, Reiman (2008) observes that the holistic community-based models can offer more social, physical mental and emotional support than the traditional pharmacy model alone. According to Reiman, such facilities respond to the often multi-faceted health needs of medicinal cannabis consumers, for whom a commonality is illness, both chronic and terminal. Public health approaches therefore need to facilitate individuals adjusting to complex health factors. Reiman (2008) notes that the social support groups, counselling and the entertainment services offered in California, can alleviate depression and facilitate social integration, a significant outcome given the often compounding needs of chronically ill consumers. Arguments have started to be made as to how the UK’s growing Cannabis Social Clubs could develop their model therapeutically, and how both the Dutch pharmacy model and the social club model could be integrated to allow for a holistic, broader and a more interconnected approach to health (Bone, 2014).

A final failure of the UK judiciary to embrace a broader conception of health is through their implied concern that the individuals in *Quayle* may have derived pleasure from their cannabis use, as opposed to it solely relieving them of pain (*Quayle*, para. 77). Rather than accepting pleasure as a valued and legitimate health outcome – in line with the WHO’s broad definition of health, encompassing the idea of ‘complete […] well-being’ – pleasure is often negated or frowned upon within the drug policy sphere (Holt & Treloar, 2008). In reality, pleasure is what motivates most, if not all, mammalian behaviour (Coveney & Bunton, 2003; Mackenzie, 2011). Thus, it is important to account for pleasure as well as harm in any judicial balancing exercise and to generally theorise a place for this notion, which various harm reduction and public health initiatives across the globe are beginning to do (Bunton & Coveney, 2011). The Chicago Recovery Alliance observes a notable increase in safer injecting practices when service users are informed that they will maximise their pleasure through injecting at a certain angle, as opposed to when the organisation solely informs them that injecting at a certain angle would limit their chances of damaging a vein (Scott, 2013). Winstock also explores the relationship between harm reduction and pleasure through generating *‘*The Highway Code: A Guide to Safer More Enjoyable Drug Use’ from the ‘Global Drug Survey’, in order to maximise the benefits and to minimise the risks derived from psychoactive substances (Global Drug Survey, 2014). Race (2009, p. ix) observes that pleasure remains a contentious issue within the drug policy field since ‘taking drugs for pleasure would appear to transgress the moral logic of ‘restoring health’ that guarantees their pharmaceutical legitimacy’. Regardless, commentators are beginning to challenge the biomedical hegemony of psychoactive research, through embracing the idea of ‘entheogenic therapy’, ‘benefit maximisation’ and ‘pleasure’ more generally into our developing conceptions of health and well-being (Blainey, 2015; Mackenzie, 2011; Tupper, 2008). Tupper’s concept of benefit maximisation feeds into reshaping understandings of medicinal cannabis consumption**.** Both the social club and pharmacy regulatory models encourage vaporising cannabis as a pleasurable and safer delivery method, which positively builds upon global public health campaigns to recognise tobacco use as a health hazard (WHO, n.d). In California, female medicinal cannabis patients can now purchase ‘Foria’, a therapeutic cannabis oil ‘designed to enhance female pleasure’ (Foria, 2014 http://foriapleasure.com/pages/about-us). The Aphrodite Group, a collective of caregivers and patients, responsible for its development, states ‘that health and pleasure are naturally inseparable’. Additionally, Chapkis (2007) highlights that for patients coming to terms with a terminal illness, the high itself and the elevation of mood they can obtain from cannabis, particularly if they are in chronic or untreatable pain, can actually be therapeutic.

## Conclusion

The global prohibition of cannabis cultivation, supply and possession might be considered to present an insurmountable barrier to the full exploration of the therapeutic potential and public health benefits of medicinal cannabis consumption. Despite this, examples of innovative approaches have emerged around the world, although some jurisdictions, notably the UK, have provided less fertile ground for them to flourish in. We have argued in this paper that the lens of human rights provides a fruitful perspective for addressing the power dynamic between state and individual which underpins the medicinal cannabis issue, by recognising a broader idea of what constitutes ‘health’. Narrow and externalist conceptions of health – as deployed in the *Quayle, Oakland Cannabis Buyers’ Cooperative* and *Gonzales* cases – fail to acknowledge adequately the human right to health and undermine public health goals by taking a parochialist approach to the issue of medicinal cannabis use. A more expansive conception of health, in contrast, appreciating both internalist and externalist views, could lead to a public heath approach which more effectively balances individual and collective interests. Through employing a human rights perspective, the paper seeks to open a dialogue to rethink drug policies in a more thoroughgoing and potentially radical way, so that a more fully public health approach to the issue of the therapeutic use of cannabis can be developed which is not constricted by the drug prohibition paradigm.
